# Boundary Objects as Dialogical Learning Accelerators for Social Change in Design for Health: Systematic Review

**DOI:** 10.2196/31167

**Published:** 2022-02-03

**Authors:** Gijs Terlouw, Derek Kuipers, Lars Veldmeijer, Job van 't Veer, Jelle Prins, Jean-Pierre Pierie

**Affiliations:** 1 NHL Stenden University of Applied Sciences Leeuwarden Netherlands; 2 Medical Faculty Lifelong Learning, Education & Assessment Research Network, University Medical Center Groningen, University of Groningen Groningen Netherlands; 3 Research Group Serious Gaming, NHL Stenden University of Applied Sciences Leeuwarden Netherlands; 4 Research Group Digital Innovation in Healthcare and Social Work, NHL Stenden University of Applied Sciences Leeuwarden Netherlands; 5 University Medical Center Groningen, University of Groningen Groningen Netherlands; 6 Post Graduate School of Medicine, University Medical Center Groningen, University of Groningen Groningen Netherlands; 7 Department of Surgery Medical Center Leeuwarden Leeuwarden Netherlands

**Keywords:** boundary objects, health, innovation, design, systematic review

## Abstract

**Background:**

Boundary objects can add value for innovative design and implementation research in health care through their organizational focus and the dynamic structure between ill-structured and tailored use. However, when innovation is approached as a boundary object, more attention will need to be paid to the preimplementation phase. Research and design thinking pay attention to the preimplementation stage but do not have a social or organizational focus per se. The integration of boundary objects in design methodologies can provide a more social and organizational focus in innovative design projects by mapping out the mechanisms that occur at boundaries during design. Four dialogical learning mechanisms that can be triggered at boundaries have been described in the literature: identification, coordination, reflection, and transformation. These mechanisms seem suitable for integration in innovative design research on health.

**Objective:**

Focusing on innovation in health, this study aims to find out whether the different learning mechanisms can be linked to studies on health innovation that mention boundary objects as a concept and assess whether the related mechanisms provide insight into the stage of the design and implementation or change process.

**Methods:**

The following 6 databases were searched for relevant abstracts: PubMed, Scopus, Education Resources Information Center, PsycINFO, Information Science and Technology Abstracts, and Embase. These databases cover a wide range of published studies in the field of health.

**Results:**

Our initial search yielded 3102 records; after removing the duplicates, 2186 (70.47%) records were screened on the title and abstract, and 25 (0.81%) papers were included; of the 13 papers where we identified 1 mechanism, 5 (38%) described an innovation or innovative project, and of the 12 papers where we identified more mechanisms, 9 (75%) described the development or implementation of an innovation. The reflective mechanism was not identified solely but was present in papers describing a more successful development or implementation project of innovation. In these papers, the predetermined goals were achieved, and the process of integration was relatively smoother.

**Conclusions:**

The concept of boundary objects has found its way into health care. Although the idea of a boundary object was introduced to describe how specific artifacts can fulfill a bridging function between different sociocultural sites and thus have a social focus, the focus in the included papers was often on the boundary object itself rather than the social effect. The reflection and transformation mechanisms were underrepresented in the included studies but based on the findings in this review, pursuing to trigger the reflective mechanism in design, development, and implementation projects can lead to a more fluid and smooth integration of innovation into practice.

## Introduction

### Background

The concept of boundary objects was introduced in 1989 by Star [1] to describe how specific artifacts can fulfill a bridging function between different sociocultural sites. Over the past decades, there has been more interest in boundaries, boundary crossing, and boundary objects [2-6]. The idea of boundary objects was initially framed to facilitate constructive cooperation between sites or social systems without consensus [7]. This organizational feature of boundary objects can be of great value in health care innovation, where the promise of innovations outweighs their actual impact [8-13]. Owing to many different stakeholders and parties in health care with different needs and goals, the implementation of innovations in health care practice is complex [14]. Many frameworks on innovation and implementation pursue consensus [15-21], mainly from a monodisciplinary approach, where at some point, all parties and stakeholders must be convinced that an innovation is of added value from a specific viewpoint. Within disciplines, this can be feasible, but across disciplines, this is often challenging. Boundary objects offer a different perspective on this issue. Boundary objects ideally address the needs of each stakeholder group and aim to contribute to the goals of all stakeholders involved, even if they do not pursue the same goal. This also means that different stakeholders can interpret a boundary object differently, something that Star [7] calls *interpretive flexibility*. However, not striving for consensus but identifying and addressing needs on the front end requires a fundamentally different approach. Much more attention will need to be paid to the preimplementation phase, which is seldom included in frameworks [22]. The design discipline is a discipline par excellence that pays attention to precisely this phase.

Design research and design thinking are increasingly finding their way into the health care sector as appropriate methodologies of responding to a world with more open, complex, and increasingly networked problems. Design holds the promise of offering suitable strategies for complex problems and actively involves stakeholders during the development and implementation of innovation [23,24]. Design as a discipline already has a long history in the development of medical devices but is now broadening its scope in shaping the future of health care [25-28]. Owing to different causes, the worlds of health care and design are converging. In health care, there is a shift in focus toward patient experience and values, increasing the quality of life and patients’ participation in care and treatment [29,30]. In the design discipline, developments toward phenomena such as experience design [31], value-sensitive design [32], and people’s involvement in design through participatory design [33-35] seem to have a good fit with the shifts in the focus of health care. The focus of emerging design disciplines on innovation, transformation, and services within organizations [36] can also solve implementation and adoption problems in innovation. However, many frameworks or models that provide insight into shaping the design process focus more specifically on the steps, methods, or guide points essential for developing an artifact [37-42] and less on shaping the process of change.

### Boundary Objects Within Design Research

#### Overview

The concept of boundary objects has also been applied in different studies on design and product development [43-45]. However, the focus is often too specific on 1 element of boundary objects: interpretive flexibility. Other elements mentioned by Star [7] are the structure of informatics, work process needs and arrangements, and the dynamic between the ill-structured and more tailored uses of the object [1,7,46]. In citations, the aspect of interpretive flexibility is overrepresented: “boundary objects almost became synonymous with interpretive flexibility” [7]. Nevertheless, in design, the interest in interpretative flexibility as a feature of boundary objects is sensible. By developing and testing concepts and prototypes with stakeholders, a lot can be learned regarding the product or idea during development, primarily through the interpretation of end users. However, the more organizational side of boundary objects to let people work together constructively and the focus of boundary objects in changing organizations can be of added value, especially in the embedding and adoption of innovation in practice. In the life cycle of boundary objects, Star [7] describes boundary objects’ role in the organizational nature as a back-and-forth movement between ill-structured and well-structured [7]. In this life cycle, parallels can be drawn with an innovation or design life cycle, a back-and-forth movement from the emergence of a complex problem in an ill-structured context to the development of a solution that ideally leads to integration in the context where a new and clear structure occurs.

Both boundary objects—through their organizational focus and the dynamic structure between ill-structured and tailored use [7]—and design—which, in complex settings, requires managing and moving multiple stakeholders from the problem to the solution space [47]—might be able to guide innovative transformations in health care. However, frameworks aimed at adoption and implementation rarely pay attention to the development process [22]; frameworks aimed at design are often more product oriented.

A possible starting point to provide more social focus during the application of boundary objects in the development and transformation of innovation can be found in a systematic review by Akkerman and Bakker [3], who described four dialogical learning mechanisms that can take place at boundaries: identification, coordination, reflection, and transformation. The mechanisms are similar to interrelational forms of boundary work that Langley et al [48] reported to describe organizational work: competitive boundary work can be linked to identification, collaborative boundary work can be related to coordination, and configurational boundary work seems synonymous with transformation. Although the initial focus of the 4 mechanisms of Akkerman and Bakker [3] was mainly on education, they seem to fit with well-known focus areas in design.

#### Identification

The identification mechanism is about learning what the diverse practices are to each other [3]. Typically, in identification processes, the boundaries are encountered, reconstructed, or reinforced. The identification mechanism is not necessarily related to overcoming discontinuities. The strategy to enact this process is something that designers often perform in the first phase of research. Many design research thinking or design research projects start with a phase or focus, such as *empathy* [49-51], *discover* [52-54] or *assess user needs, analyze content, and context* [55-57], in which, inter alia, user needs are identified. In design, this phase is essential. It allows designers and researchers to comprehend the situations and perspectives of others [58]. During this phase, methods such as *empathy maps* [59], *personas* [60,61], and *a day in the life* [62] are used to identify visible and invisible components that define the stakeholders’ identities and needs, which fits the dialogical learning mechanism of identification. The stakeholders and designers are reinforced in their roles and (professional) identities; boundaries are encountered, reconstructed, or reinforced but not overcome in this phase.

#### Coordination

Dialogical learning mechanism coordination is mainly about creating cooperative and constructive exchanges between practices, even without consensus [3]. This description is the closest to the concept that Star [1,7,46] originally presented. It is crucial for design teams to work together effectively and constructively in the design discipline, even if the backgrounds and practices differ. Within the design, multilayered interactions can occur, and through the development of co-design practices, users can become active participants in design projects and processes [63,64]. There is a wide variety of methods in design to facilitate constructive collaboration between practices in health innovation, such as *hackathons* [65-67], *future workshops* [68,69], and other creative participatory design methods [70-72]. The potential of the coordination is in (temporarily) overcoming boundaries and getting to know each other, not in reconstructing them. This usually fits the design stage where there are no objectives formulated yet; the problem still needs to be defined, and the co-ownership of different stakeholders is desirable.

#### Reflection

The dialogical learning mechanism reflection emphasizes the role of boundary crossing and boundary objects in realizing, clarifying, and exchanging differences between practices [3]. Reflection is about expanding perspectives through perspective taking and perspective making. Together with the identification mechanism, the reflective mechanism focuses mainly on meaning-oriented learning processes. Once enacted, the reflective mechanism results in an expanded set of perspectives that inform future practices. Within the design discipline, and as a designer, reflection is essential. Schön [73] describes the creative process as a continuous process of reflection in action. Following the theory by Schön [73], designers enfold a continuum of activity by reflecting and acting within a new situation. The designer and stakeholder reflections help to frame and move the problem toward the common ground. Both the designer and the parties at stake continuously learn and reflect in a dialogical way. This dialogical learning is essential within participatory design, as participatory design sees people as the real experts of domains and experiences [74]. The notion of design of Simon [75] that design attempts to change existing situations into preferred ones transcends the designer’s role in a complex setting; the whole network of stakeholders is necessary to get to the preferred situation. In a complex context, the preferable situation is inherently multileveled; therefore, it seems essential that different stakeholders reflect and expand their perspectives to formulate constructive objectives and inform future practice. A new *change space* might occur through dialogic reflection, where there is room for new ways of framing the problem by highlighting its paradoxes and eventually generating different possible solutions [76,77]. The reflective learning mechanism is often enacted by proposing or evaluating an intervention [3], which fits the nature of design by testing and assessing specific ideas, visualizations, concepts, and prototypes. The focus on social change and the emergence of a shared mental model regarding perspective making and perspective taking, informing future practice, might be a specific addition to the design process direction, providing social support to frame and reframe the problem. Unlike the identification mechanism, reflection is about overcoming boundaries and shaping future practice, where stakeholders are aware of the different perspectives resulting from perspective taking and perspective making.

#### Transformation

The dialogical learning mechanism transformation is about collaboration and the development or codevelopment of new practices [3]. The transformation mechanism is characterized by the process from a shared awareness of a problem to the development and, eventually, the crystallization of a new and maintainable setting. The ill-structured context becomes one where the innovation is characterized by tailored use. The emergence of a new context such as this is often the ultimate goal of both innovation and design. In the transformation phase, a shared problem space is necessary to get the whole network moving. Therefore, dialogical reflection in the system seems essential to advance a network to the transformation or change space.

Although there are design activities at the intersection of learning mechanisms, the learning mechanisms seem to be suitable for evaluating the degree of social change during the design process. In addition to the continuous reflection on the development of the product and the frame, it can be of added value to reflect on the social process early in the design process, using learning mechanisms to increase the chances of integration and adoption.

#### Aims

Focusing on innovation in health, this study aims to find out whether the different learning mechanisms can be linked to studies on health innovation that mention boundary objects as a concept and assess whether the related mechanisms provide insight into the stage of the design and implementation or change process.

## Methods

### Databases and Search Strategy

The following six databases were searched for potentially relevant abstracts: PubMed, Scopus, Education Resources Information Center, PsycINFO, Information Science and Technology Abstracts, and Embase. These databases cover a wide range of published research in the field of health care. They were selected after several trial searches in various databases and after consultation with an information specialist in health science. The terms that were used for the search in PubMed are presented in Textbox 1.

Owing to differences in search engine functionality, the method by which terms were entered differed per database. A complete overview of the terms is included in Multimedia Appendix 1. Searches included papers published between 1989, when Star [1] introduced the concept of boundary objects, and September 2020. Before the definitive search, we performed 3 trial searches with different terms to reduce the possibility of missing relevant studies. We conducted a definitive search on September 23, 2020. We followed the PRISMA (Preferred Reporting Items of Systematic Reviews and Meta-Analyses) guidelines [78] as much as possible to report this review.

**Search terms used for relevant abstracts in PubMed.**

**Search terms**

- (boundary object*[tiab] OR boundary cross*[tiab]) AND (*“Diffusion of Innovation” [Mesh]* OR *“Organizational Innovation” [Mesh]* OR *“Research” [Mesh]* OR *“Interdisciplinary Communication” [Mesh]* OR *“Negotiating” [Mesh]* OR dialogic*[tiab] OR *participatory[tiab]* OR learn*[tiab] OR innovat*[tiab] OR design*[tiab] OR develop*[tiab] OR research*[tiab] OR interdisciplin*[tiab] OR cross disciplin*[tiab] OR multidisciplin*[tiab] OR negotiat*[tiab] OR mediat*[tiab])

### Study Selection and Inclusion and Exclusion Criteria

We included studies that discussed boundary objects or innovations in health. We included only original reports or papers that (1) mentioned boundary objects, (2) involved an empirical study, or (3) otherwise focused on a newly developed or implemented innovation. Papers meeting these criteria were selected for full-text screening.

The following exclusion criteria were used for full-text screening: (1) non–peer-reviewed papers such as abstracts, conference posters, or trade journals; (2) papers with full text not available; (3) papers in languages other than English; (4) monographs or short reports; and (5) papers with not sufficient information in the abstract.

### Screening Process

After removing the duplicates, the papers were screened based on title and abstract using Rayyan [79]. A total of 2 reviewers (GT and DK) independently reviewed all titles and abstracts, who were double-blinded for relevance with the formulated inclusion and exclusion criteria. Papers were only included on the agreement of both GT and DK, where a plausible argumentation for consideration of inclusion always led to inclusion. Full-text papers were retrieved after this step. During the full-text screening phase, the first 20% of the papers were randomly selected and double-blind reviewed by 2 reviewers (GT and LV). After this scan, no disagreements occurred about inclusion or the identified mechanisms. Then, the main reviewer (GT) reviewed the other included papers for a full-text reading.

## Results

### Search Results

**Figure 1:**
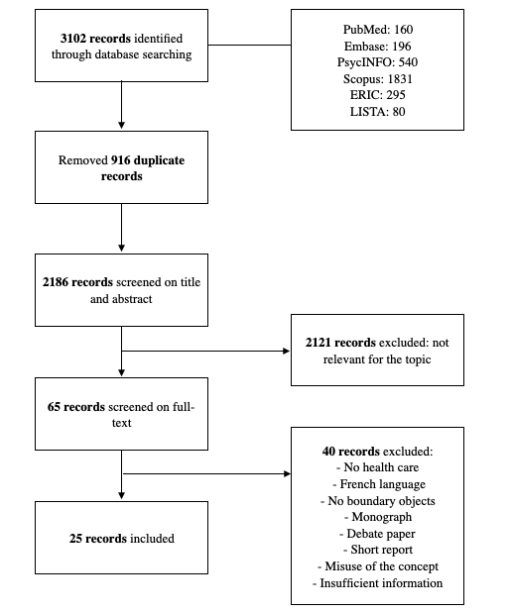
Flowchart of the selection process. ERIC: Education Resources Information Center; LISTA: Library, Information Science and Technology Abstracts.

Our initial search yielded 3102 records. Of the 3102 records, after removing the 916 (29.53%) duplicates, 2186 (70.47%) records were screened based on their titles and abstracts. Next, of the 65 records, we screened the full text, leaving 25 (38%) papers for inclusion (see Figure 1 for a flowchart of the results in the different selection stages). In both stages, there was a consensus between the reviewers on both the inclusion and analysis of the papers.

### General Findings

The studies included in this systematic review had varied study designs and topics. Table 1 presents the study designs, topics, and characteristics. All the included articles were published after 2008.

We studied full-text papers on applying the concept of boundary objects; whether this concept was used to describe daily life situations or situations where there was innovation either in its development, implementation, or postimplementation stage; and if ≥1 dialogical learning mechanisms could be identified. In the following section, we categorize the papers based on their mechanisms and the situations they applied to.

**Table 1:** Papers included in the systematic review.

Study	Title	Identified learning mechanisms	Phase
Nielsen and Mengiste, 2014 [80]	Analyzing the diffusion and adoption of mobile IT^a^ across social worlds	Identification Transformation	Postimplementation
Lambert et al, 2019 [81]	Antimicrobial resistance, inflammatory responses: a comparative analysis of pathogenicities, knowledge hybrids and the semantics of antibiotic use	Identification	Operational
Kajamaa, 2011 [82]	Boundary breaking in a hospital: expansive learning between the worlds of evaluation and frontline work	Identification Coordination Reflection Transformation	Development and implementation
Bjørn et al, 2009 [83]	Boundary factors and contextual contingencies: configuring electronic templates for health care professionals	Identification	Development and implementation
Sajtos et al, 2018 [84]	Boundary objects for institutional work across service ecosystems	Identification Coordination Reflection Transformation	Implementation
Jensen and Kushniruk, 2016 [85]	Boundary objects in clinical simulation and design of eHealth	Identification Coordination Reflection Transformation	Development and implementation
Sampalli et al, 2011 [86]	Clinical vocabulary as a boundary object in multidisciplinary care management of multiple chemical sensitivity, a complex and chronic condition	Coordination	Operational
Sampalli et al, 2009 [87]	Boundary objects in the multidisciplinary care management of chronic conditions: multiple chemical sensitivity	Coordination	Operational
Fox, 2011 [88]	Boundary objects, social meanings and the success of new technologies	Transformation	Postimplementation analysis
Jentoft, 2020 [89]	Boundary-crossings among health students in interprofessional geropsychiatric outpatient practice: collaboration with elderly people living at home	Coordination Reflection	Project
Håland et al, 2015 [90]	Care pathways as boundary objects between primary and secondary care: experiences from Norwegian home care services	Coordination Reflection Transformation	Development and implementation
Sajtos et al, 2014 [91]	Case-mix system as a boundary object: the case of home care services	Coordination	Development
Islind et al, 2019 [44]	Co-designing a digital platform with boundary objects: bringing together heterogeneous users in health care	Identification Coordination Transformation	Development
Meier, 2015 [92]	Collaboration in health care through boundary work and boundary objects	Coordination	Operational
Williams et al, 2008 [93]	Human embryos as boundary objects? Some reflections on the biomedical worlds of embryonic stem cells and preimplantation genetic diagnosis	Identification	Operational
Keshet and Popper-Giveon, 2013 [94]	Integrative health care in Israel and traditional Arab herbal medicine: when health care interfaces with culture and politics	Identification Coordination Reflection Transformation	Operational
Marabelli et al, 2017 [95]	Knowledge sharing and health care coordination: the role of creation and use brokers	Identification Coordination	Development and implementation
Islind and Snis, 2017 [96]	Learning in home care: a digital artifact as a designated boundary object-in-use	Identification Coordination Transformation	Development and implementation
Isah and Bystroöm, 2020 [97]	The mediating role of documents: information sharing through medical records in health care	Coordination	Operational
Gregory et al, 2014 [98]	Patient experiences of diabetes eHealth	Coordination	Postimplementation
Stewart and Watson, 2019 [99]	A Sociotechnical history of the ultralightweight wheelchair: a vehicle of social change	Transformation	Postimplementation
Melo and Bishop, 2020 [100]	Translating health care research evidence into practice: the role of linked boundary objects	Coordination Identification	Postimplementation and operational
Mengiste and Annestad, 2013 [101]	Understanding the dynamics of learning across social worlds: a case study from implementing IS^b^ in the Ethiopian public health care system	Identification Coordination	Development
McLoughlin et al, 2016 [102]	Doing infrastructural work: the role of boundary objects in health information infrastructure projects	Coordination	Development and implementation
Terlouw et al, 2020 [57]	Design of a digital comic creator (It's Me) to facilitate social skills training for children with autism spectrum disorder: design research approach	Coordination Reflection Transformation	Development

^a^IT: information technology.

^b^IS: information system.

### Identification

Of the 25 papers, 2 (8%) [81,93] described a medical term as a boundary object to identify different medical terms’ interpretations over different contexts or disciplines. Lambert et al [81] approached terms such as *infection*, *antibiotics*, and *inflammation* as boundary objects. Williams et al [93] conceptualized embryos to find out how they were decontextualized and recontextualized within and between 2 different cultural systems. In both studies, existing beliefs were reinforced, and the studies aimed to identify local differences by applying the identification mechanism in a noninnovation context.

In 8% (2/25) of the papers, the identification mechanism was deduced in more innovative projects. Nielsen and Mengiste [80] described in their study an analysis of the adoption of mobile information technology innovation for home care. The innovation functioned as a boundary object on the level of influential stakeholders (ministry, local government, and managers). Managers and care workers revealed different interpretations of the technology’s value and potential, resulting in resistance and tension. The technology seemed to reinforce existing differences between activity systems at the level of managers and care workers. The authors proposed a bottom-up approach and more involvement of end users in the future. This technology seemed to reinforce existing differences, triggering the identification mechanism.

Bjørn et al [83] described “conflicting perspectives between standardization and reconfiguration embedded within hospital information systems (HIS) design activities.” In their study, the authors considered an electronic triage and tracking system as a boundary object. Users indicated whether they could work with the system and how. This led to adjustments and reconfigurations in the system and, presumably, better adoption of the system but not to, for example, perspective taking between different groups. Differences were primarily sought for user groups to use the system optimally by having them respond to the system, thus using the identification mechanism in this study in a constructive way to retrieve specific input.

### Coordination

In 16% (4/25) of the papers, we identified the coordination mechanism to facilitate cooperation in daily practice. Approximately 50% (2/4) of these papers presented controlled clinical vocabulary to facilitate and coordinate collaboration between different professionals [86,87]. Approximately 50% (2/4) other studies make use of data or narratives to facilitate multidisciplinary cooperation. Meier [92] described an ethnographic study of 2 hospital wards. Patients’ stories, especially their narratives and patient records, formed the boundary objects to make constructive multidisciplinary work possible. A study by Isah and Bystroöm [97] focused on the role of case notes as mediating artifacts in patient care. Their study demonstrated how case notes were a source of information and an essential, enacting, and mediating part of the work itself. The case notes seemed to be multipurpose; they served as a repository of information and knowledge and supported and mediated a plethora of the medical team’s work activities in patient care. It was evident that the case notes served as a coordinating mechanism between the participating actors. Besides facilitating and fortifying many day-to-day functions in patient care, case notes have established themselves for deliberate learning; they embody a clerkship template and enable newcomers to integrate and perpetuate the practice.

In 12% (3/25) of the papers, we identified a coordination mechanism to promote innovation. The coordination mechanism was identified in the first of 2 included papers from Sajtos et al [91], where the authors reported a case study of developing a case-mix system. Their study illustrated a process to address the diverse meanings and interests of various stakeholders to overcome communication and organizational challenges. They presented a funneling framework. A so-called boundary concept evolved through stakeholder input into a boundary object in a second step and a solution in the final step. Both principles and constraints were identified and addressed in the final solution by aligning the stakeholders’ interests. In the project, no clients were directly involved, and it remains unclear whether and how the design itself was subject to flexibility along the way or that the project was mainly about fine-tuning a design.

Gregory et al [98] evaluated a diabetes eHealth system in their study. They described the use of the system as a boundary object for developing an understanding of why the eHealth system was used in a wide variety of ways, enabling coordination over stakeholder groups. McLoughlin et al [102] reported 4 case studies of health information infrastructure projects, where they approached the health information systems as boundary objects. Two of the projects were on a regional level, and 2 projects were on a national level. In the regional projects, the boundary objects managed and facilitated collaboration in using health data for different purposes by different users. At the national level, the 2 boundary objects instantiated top-down attempts and struggled more to trigger some effects.

### Reflection

We identified no papers which merely reported the reflection mechanism.

### Transformation

The transformation mechanism was identified in 8% (2/25) of the studies. Both studies described a more extended transformational development in retrospect. Fox [88] described the development of antiseptic and aseptic environments during surgery. In a historical case study, the report assessed the innovations in surgical sterility and how boundary objects worked over time. For example, in this study, nose masks were considered boundary objects in their relationship to social meanings within communities of practice. In the conclusions, the researcher described positive and negative boundary objects and concluded the following:

Boundary objects are not merely passive vehicles that allow communication between communities of practice or knowledge but elements that encapsulate the broader social meaning of a concept, theory, technology or practice, and the underlying relations surrounding its development and adoption.

This study described the surgery profession’s transformation process, partly through boundary objects, from only a healer of disease to a healer of disease who was also a safety procurer.

The second historical perspective was written by Stewart and Watson [99], who described the development of the ultralightweight wheelchair and its social implications. As a boundary object, the ultralightweight wheelchair had a significant transformational impact on the use of wheelchairs in the daily lives of users of wheelchairs. According to the authors, the wheelchair as a boundary object provided many insights through various interpretations of the artifact. It reflected views about users of wheelchairs and disability more generally and how the ultralightweight wheelchair as a boundary object seemed to manifest power relations between the diverse communities it engaged.

### Multiple Mechanisms

#### Overview

Of the included 25 studies, in 12 (48%) studies, we identified ≥1 mechanism. Of these 12 papers, 3 (25%) focused more on structuring *everyday* practices, 9 (75%) identified multiple mechanisms that focused explicitly on developing or implementing a new tool, 6 (50%) were concentrated mainly on professionals and professional collaborations, and 3 (25%) actively focused on processes involving clients or patients at the center.

#### Studies Structuring Everyday Practice

Jentoft [89] described in her research boundary-crossing activities among physical therapy and medical students in interprofessional geropsychiatric outpatient practice. In the study, the students visited older clients living at home on 2 occasions. On the basis of these visits, the students considered suitable interventions for clients to enhance their quality of life, health outcomes, and well-being. After that, students wrote a health record to document their professional and interprofessional views on the cases. The health record and its content served as the boundary object during the study. The health record itself coordinated collaboration between the different disciplines by helping them plan examinations and establish a relationship with the client. The health record content enhanced reflection and negotiation and ensured that students understood the other’s (professional) perspective better. In conclusion, the boundary objects led to more effectiveness and improved evaluation quality through better interprofessional collaboration, and students became more knowledgeable about what others and other professions did in practice.

Keshet and Popper-Giveon [94] explicitly used the learning mechanisms of Akkerman and Bakker [3] to describe the integration of local traditional medicine within complementary medicine. Their article aimed to contribute to the “contemporary critical debate in medical anthropology concerning medical pluralism and integrative medicine” by highlighting the exclusion of traditional medicine. Through ethnographic fieldwork, they focused on a group of integrative physicians who had recently begun integrating conventional herbal medicine. By conceptualizing traditional medicine as a boundary object, they attempted to bridge professional gaps between biomedicine, complementary medicine, and traditional medicine. Their study showed that using herbal medicine as a boundary object helped overcome barriers and provided a window for dialog and learning at different levels.

Melo and Bishop [100] described a fall risk scale combined with a pink wristband as a boundary object. The pink wristband was used to signal patients with a high fall risk, measured by a falling score. Communicating the meaning of the pink wristband to other hospital staff improved the coordination and facilitation of work organization around persons with higher fall risk.

#### Studies Focusing Mainly on Professional Collaboration

This section describes the 24% (6/25) of papers where multiple identified mechanisms focused on developing or implementing a new tool to enhance or trigger professional collaboration. In 33% (2/6) of these papers, we discovered the identification and coordination mechanisms. Marabelli et al [95] described the development and implementation of a summary medical note (the single point of care]) carried by parents between the specialists involved in their child’s care. Their paper described the single point of care as a boundary object with coordinative mechanics to enhance and facilitate communication between different stakeholders. In the predevelopment phase, parents had an important role in identifying and addressing the problem. The interviews and sessions had the characteristics to trigger the identification mechanism. In their analysis, the authors demonstrated that “the SPOC’s effectiveness can be understood by looking at the combined roles of boundary objects and human brokers*.*”

Mengiste and Annestad [101] reported a case study on the implementation of information systems in the Ethiopian public health care system. The paper analyzed how this software functioned as a boundary object. They found that the software did not just facilitate cooperation among the actors; the software as a boundary object also had a role in *bringing the existing differences to the foreground*, applying the identification and coordination mechanism. In addition to the software, which the authors explicitly called a boundary object, many sessions, workshops, test sessions, and prototypes were described, which also had the characteristics of boundary objects.

In the paper by Håland, Røsstad, and Osmundsen [90], coordinating and reflection mechanisms were identified. They studied the development, introduction, and use of “a care pathway across healthcare levels focusing on older home-dwelling patients in need of home care services after hospital discharge.” Their study explored how care pathways can use the concept of boundary objects in translation between specialist health care services and home care services. Interviews with the project participants found that the “response to existing needs, local tailoring, involvement, and commitment are all crucial for the care pathway to function as a boundary object.” Furthermore, they described that the artifact could “push boundaries just as much as it can be used as a tool for bridging across them” [90]. By introducing the care pathway system early, as an idea, to different stakeholders, they could address specific needs in the system, resulting in better integration. The introduction of the care pathway system led to collaboration and coordination among organizations, better understanding, reflection on different perspectives (eg, between home care workers and hospital care workers), and new ways of working in transformed activity systems.

In 12% (3/25) of the papers focusing on professionals and professional collaboration, we identified all 4 mechanisms. Jensen and Kushniruk [85] presented a case study on a participatory design process of electronic documentation templates for nurses, which they used for patient assessment:

Clinical simulation was used as a boundary object and thereby achieved mutual clinical agreement on the content. By using clinical simulation, knowledge was transferred and transformed between the different communities of practice to support gaining a shared understanding.

This was mainly to overcome organizational barriers. As they presented in their case study, the clinical simulation might have helped form “shared mental models and shared understanding of user requirements, work practice and organizational requirements” within an innovation project. The boundary objects approach helped analyze vital issues and triggered a reflective approach to improving solutions. This case study showed that the adoption and acceptance of new technology might be significantly improved by leading end users and other important stakeholders within the organization through all mechanisms.

Sajtos et al [84] introduced the concept of boundary objects to facilitate institutional work across different ecosystems through a case-mix system. They conducted qualitative interviews with three key actors—funding agency, service provider, and clinicians—to identify these actors’ views on the nature of home-based support services and their impact as a boundary object within the implementation of a case-mix system. Their analysis was based on three interviews: 1 before introducing the case-mix system, 1 just after the introduction, and 1 after the introduction. This provided a comprehensive view of an implementation process in which the concept of boundary objects was juxtaposed. The prephase mainly reported data reflecting the identification mechanism, where actors defined themselves mainly through differences between them. After the introduction, the case-mix system as a “boundary object enabled the actors to reframe and theorize about their idiosyncratic meanings of healthcare provision and embrace some new aspects.” This led to perspective making and reframing of their own views to eventually use a jointly operated system by introducing new routines and practices that identified the reflection and transformation mechanisms. The reported study seemed to reflect the fluid implementation process by using the concept of boundary objects. The study did not report any adjustments made to the artifacts themselves because of the activated mechanisms or design rationale.

Kajamaa [82] reported a case study on the innovative creation process of an assessment tool in which nurses and quality controllers participated. Through different steps, the diverse needs of nurses and quality officers were reinforced and addressed. Both stakeholder groups collaborated on developing a tool, reflected on designs that led to perspective making and perspective taking, and finally started the implementation process together. The different *in-between* versions of the tool acted as boundary objects. During implementation, 2 events occurred. The first event resulted from new circumstances, which were illustrative of solutions: problems are not static. This event was overcome during the project. The second event led to a breach of trust between the stakeholder groups and, thus, to the project’s end. The initially overcome differences between the stakeholder groups were reinforced again by triggering the identification mechanism in a different way than the first time.

#### Studies Involving Clients or Patients

Of the 25 studies, 3 (12%) actively focused on processes involving clients or patients at the center. In these studies, clients or patients actively participated, and 3 mechanisms were identified. Islind et al [44] applied the concept of boundary objects in a co-design project for a digital platform at a clinic that supported cancer patients in their struggles with treatment-induced illnesses. This paper explicitly explored the functions that boundary objects can have in a design process and how they were engaged in the different design phases. Islind et al [44] described the following three types of boundary objects: narratives as open boundary objects in the first phase, metaphorical boundary objects as semiopen boundary objects in the second phase, and structured boundary objects in the third phase. Although the focus was more on the boundary objects’ different characteristics during a design project, implicitly, the mechanisms that the boundary objects enacted were also described. The first type of boundary objects—the narratives—seemed to trigger the identification mechanism to better understand the user groups:

The narratives, in the forms of patient stories, played a central role for understanding the patient group and the healthcare professionals as the needs of both user groups needed to be accommodated for.

In a way, the narrative became the container of the essence of being a patient.

In what Islind et al [44] called the *metaphorical phase*, boundary objects facilitated conversation, collaboration, and consultation among stakeholders, aligning with the coordination mechanism. In the structured phase, the boundary objects matured more as prototypes. They triggered a conversation about the platform’s future functions, aligning with the first signs of transformation. Their conclusion stated the following:

Designing with boundary objects might slow down the design process initially but actually speed up the programming process as fewer aspects will come as a surprise during the software development when everything has been negotiated thoroughly on beforehand.

In a study by Islind and Snis [96], the focus was on developing and deploying a mobile health (mHealth) artifact for groceries in home care settings. An mHealth artifact “was tested to see how the quality of home care work practice was enhanced and changed.” The mHealth artifact was presented in this paper as a boundary object. The authors presented the artifact as a designated boundary object and a boundary object in use. As a boundary object, the mHealth artifact triggered different mechanisms. In conversations, the tool reinforced the identity of older adults. For example, they realized how long they had not been to a grocery store. From the older adults’ perspective, the boundary object functioned as “a substitute for their previous buying groceries.” From the caregivers’ perspective, the boundary object was designed to “support a more efficient working process,” triggering both coordinate and transformational mechanisms. The time earlier spent in the grocery store now went to the older adults, leading to more caregiving quality in praxis. The mHealth tool was described as follows:

Mediating tool for a deepened caring conversation-in-practice where interactions and realizations generate new emerging properties and opportunities. The boundary object-in-use proved to function as a conversation starter where the use facilitated fruitful conversations between the elderly and caregivers about new aspects of grocery shopping.

In addition, new diet and “nutrition explorations were interpreted and negotiated via their evolved conversation*.*” This reshaping of the home care practice affected the caregivers’ role, “evolving into a more meaningful caretaking and nurturing role.”

Terlouw et al [57] described the development of a digital comic creator for children with an autism spectrum disorder. The digital tool was approached during the process and designed as a boundary object, aiming to connect the different stakeholders’ objectives. This led to an inclusive design and triggered reflection and transformation learning mechanisms along the way.

## Discussion

### Boundary Objects in Health

This review shows that the concept of boundary objects has found its way into health care. The use of the concept has been growing since 2008, with a significant number of papers describing boundary objects from the past 5 years. In the reviewed studies, we see that boundary objects are mainly used to shape and organize multidisciplinary work, close to the original explanation of Star and Griesemer [46], or to surface differences in, for example, interpretation of a concept from different contexts or disciplines. In the 25 papers, 38 mechanisms were identified, of which 15 (39%) were coordination mechanisms, and 10 (26%) were identification mechanisms. In addition to the organizing and performative effect, boundary objects can reinforce boundaries and create conflicts. In addition to the proposition by Star and Griesemer [46], Oswick and Robertson [103] referred to *barricades and mazes* that generate conflict and reinforce boundaries and existing differences, something that Langley et al [48] also described as part of competitive boundary work. This can be an opposing and perhaps unwelcome side of the identification mechanism in terms of change management. In the study by Kajamaa [82], we saw this effect. First, in what seems a fluid development and implementation process, they applied the identification mechanism to identify different stakeholders’ needs. After implementation, 1 event led to a breakdown of trust between stakeholders, which led to the project’s withdrawal. After this event, the boundary object was primarily used to name the significant differences between stakeholders and compete for a position without the other.

Although the concept of a boundary object was introduced to describe how specific artifacts can fulfill a bridging function between different sociocultural sites [1] and thus, have a social focus, the focus in the included papers was often on the boundary object itself rather than the social effect. Various labels were given to boundary objects in different studies, which described a more designerly process for an artifact. In the different included and excluded studies, we saw a differentiation between *designated boundary objects* and *boundary objects in use* [96,104,105]. This differentiation can be seen as parallel to the design research process. An artifact or solution continues to take shape and is developed in small steps from prototype to *object in use*. The analogy can also be made by applying a boundary object from a more ill-structured to a more well-structured context. In a second included paper of Islind et al [44], they described three types of boundary objects: narratives as open boundary objects in the first phase, metaphorical boundary objects as semiopen boundary objects in the second phase, and structured boundary objects in the third phase. Although again, the focus was more on the development of the object itself rather than the effects of the object in the social context, parallels can be drawn with a design process and application of the learning mechanisms in practice, as can be seen in the results.

The reflection and transformation mechanisms are underrepresented in the included studies. Of the 25 studies, 2 (8%) describe the transformative effect of boundary objects from a historical perspective [88,99], describing a long timeline of a particular development. However, it is difficult to determine the impact of the boundary object itself in retrospect as it is likely that many more variables played a role in the transformational processes. In addition, it is difficult to determine, in retrospect, whether the boundary objects were deliberately deployed for the given purpose. The reflective mechanism was the least identified in all the papers. However, in the papers in which the reflective mechanism took place [57,82,84,85,90], there was a much smoother adaptation and application of the innovation or tool afterward. There was more shared ownership of the problem and solution in the processes described and more consideration of other perspectives along the way. This reinforces the idea of reflection as an essential step in the design process, especially in a more complex setting with multiple stakeholders, needs, and interests. When these are appropriately addressed in the design through a boundary object’s focus and, simultaneously, addressed within the design, more mutual understanding arises. This leads to a natural emerging change space where everyone is willing to move forward [76,77].

On the basis of the findings of this study, for future design and implementation projects, the social focus of boundary objects can add value to innovation projects. Pursuing to trigger the reflective mechanism can lead to the benefit of more fluid and smooth integration of innovation into practice. Here, the boundary object perspective avoids the pursuit of consensus, which often proves unfeasible in complex practices with many stakeholders. The reflective mechanism creates a shared awareness that there are multiple perspectives and needs. This awareness can lead to a shared change space in which innovation can flourish.

### Strengths and Limitations

As seen in previous research [106], little attention has been paid to describing a conscious rationale for designing innovative artifacts in health care research. This makes it hard to determine the thoughts and foundations of a designed object. In this study, this fact also made it difficult to ascertain the intent behind the deployment of particular boundary objects. The effect was often identifiable; however, it was impossible to determine whether it was directed or accidental without knowing the intention. In addition, no study described what changes were explicitly made to a prototype or design after a specific stakeholder workshop or meeting. The often implicit focus on effect is evident in health care research, making it difficult for innovative design processes to get sensible insight into the design rationales of others.

Another observation was that many innovations in the included studies were more administrative systems, such as electronic patient files. These are pre-eminently systems with which different disciplines must work, and boundary objects are thus helpful; however, 12% (3/25) of studies showed that boundary objects are also of added value in research in which clients or patients have an active role. This observation raises the idea that there are still more gains that can be found by involving end users earlier in design processes.

The included papers were subject to the interpretation, discussion, and consensus of the reviewers (GT, DK, and LV). To counteract subjectivity as much as possible, papers were double-blind reviewed by 2 reviewers in the title and abstract scan (GT and DK). They were only included in the consensus of both reviewers. In the full-read phase, 20% (13/65) of the papers were double-blind reviewed by 2 reviewers (GT and LV) before they were discussed. No disagreements on inclusion occurred during the discussion.

### Conclusions

The concept of boundary objects has found its way into health care. In this review, we saw that boundary objects in health are primarily used to shape and organize multidisciplinary work or to surface differences in, for example, the interpretation of a concept from different contexts or disciplines. Although the concept of a boundary object was introduced to describe how specific artifacts can fulfill a bridging function between different sociocultural sites and thus have a social focus, the focus in the included papers was often on the boundary object itself rather than the social effect. The reflection and transformation mechanisms were underrepresented in the included studies; however, based on the findings in this review, pursuing to trigger the reflective mechanism in design, development, and implementation projects can lead to the benefit of more fluid and smooth integration of innovation into practice.

## Appendix

Multimedia Appendix 1 Search keys used per database.

## Abbreviations

mHealth: mobile health
PRISMA: Preferred Reporting Items of Systematic Reviews and Meta-Analyses
