# Supplementary material for: Boundary Objects as Dialogical Learning Accelerators for Social Change in Design for Health: Systematic Review
Source: JMIR Hum Factors. 2022 Feb 3;9(1):e31167. doi: 10.2196/31167 (PMC8855288; doi:10.2196/31167)
Supplement: Multimedia Appendix 1 [file humanfactors_v9i1e31167_app1.docx]

## Multimedia appendix 1

Searchkey per database:

Pubmed:

(boundary object*[tiab] OR boundary cross*[tiab]) AND ("Diffusion of Innovation"[Mesh] OR "Organizational Innovation"[Mesh] OR "Research"[Mesh] OR "Interdisciplinary Communication"[Mesh] OR "Negotiating"[Mesh] OR dialogic*[tiab] OR participatory[tiab] OR learn*[tiab] OR innovat*[tiab] OR design*[tiab] OR develop*[tiab] OR research*[tiab] OR interdisciplin*[tiab] OR cross disciplin*[tiab] OR multidisciplin*[tiab] OR negotiat*[tiab] OR mediat*[tiab])

Total 160

Embase:

('boundary object*':ti,ab,kw OR 'boundary cross*':ti,ab,kw) AND ('innovation'/exp OR 'research'/exp OR 'interdisciplinary communication'/exp OR dialogic*:ti,ab,kw OR participatory:ti,ab,kw OR learn*:ti,ab,kw OR innovat*:ti,ab,kw OR design*:ti,ab,kw OR develop*:ti,ab,kw OR research*:ti,ab,kw OR interdisciplin*:ti,ab,kw OR ‘cross disciplin*’:ti,ab,kw OR multidisciplin*:ti,ab,kw OR negotiat*:ti,ab,kw OR mediat*:ti,ab,kw) NOT ('conference abstract'/it OR 'conference paper'/it)

Total 178

PsycINFO

("boundary object*" OR "boundary cross*") AND (dialogic* OR participatory OR learn* OR innovat* OR design* OR develop* OR research* OR interdisciplin* OR “cross disciplin*” OR multidisciplin* OR negotiat* OR mediat*)

Total 540

Scopus

( TITLE-ABS-KEY ( "boundary object*" OR "boundary cross*" ) ) AND ( TITLE-ABS-KEY ( dialogic* OR participatory OR learn* OR innovat* OR design* OR develop* OR research* OR interdisciplin* OR "cross disciplin*" OR multidisciplin* OR negotiat* OR mediat* ) ) AND ( EXCLUDE ( DOCTYPE , "cp" ) OR EXCLUDE ( DOCTYPE , "cr" ) OR EXCLUDE ( DOCTYPE , "no" ) )

Total 1831

ERIC

("boundary object*" OR "boundary cross*") AND (dialogic* OR participatory OR learn* OR innovat* OR design* OR develop* OR research* OR interdisciplin* OR “cross disciplin*” OR multidisciplin* OR negotiat* OR mediat*)

Total 295

Library, Information Science & Technology Abstracts (LISTA)

("boundary object*" OR "boundary cross*") AND (dialogic* OR participatory OR learn* OR innovat* OR design* OR develop* OR research* OR interdisciplin* OR “cross disciplin*” OR multidisciplin* OR negotiat* OR mediat*)

Total 80
